# Identification and Quantification, Metabolism and Pharmacokinetics, Pharmacological Activities, and Botanical Preparations of Protopine: A Review

**DOI:** 10.3390/molecules27010215

**Published:** 2021-12-30

**Authors:** Wangli Huang, Lingbo Kong, Yang Cao, Liang Yan

**Affiliations:** 1 Department of Spine, Honghui-Hospital, School of Medicine, Xi’an Jiaotong University, Xi’an 710054, China; wanglihuang0107@163.com (W.H.); lbkongmo@163.com (L.K.); yaocaoyang@163.com (Y.C.); 2 Department of Orthopedics, School of Medicine, Yan’an University, Yan’an 716000, China

**Keywords:** natural products, protopine, pharmacological activities

## Abstract

Through pharmacological activity research, an increasing number of natural products and their derivatives are being recognized for their therapeutic value. In recent years, studies have been conducted on *Corydalis yanhusuo* W.T. Wang, a valuable medicinal herb listed in the Chinese Pharmacopoeia. Protopine, one of its components, has also become a research hotspot. To illustrate the identification, metabolism, and broad pharmacological activity of protopine and the botanical preparations containing it for further scientific studies and clinical applications, an in-depth and detailed review of protopine is required. We collected data on the identification and quantification, metabolism and pharmacokinetics, pharmacological activities, and botanical preparations of protopine from 1986 to 2021 from the PubMed database using “protopine” as a keyword. It has been shown that protopine as an active ingredient of many botanical preparations can be rapidly screened and quantified by a large number of methods (such as the LC-ESI-MS/MS and the TLC/GC-MS), and the possible metabolic pathways of protopine in vivo have been proposed. In addition, protopine possesses a wide range of pharmacological activities such as anti-inflammatory, anti-platelet aggregation, anti-cancer, analgesic, vasodilatory, anticholinesterase, anti-addictive, anticonvulsant, antipathogenic, antioxidant, hepatoprotective, neuroprotective, and cytotoxic and anti-proliferative activities. In this paper, the identification and quantification, metabolism and pharmacokinetics, pharmacological activities, and botanical preparations of protopine are reviewed in detail to lay a foundation for further scientific research and clinical applications of protopine.

## 1. Introduction

At present, natural products are under focus in the medicine, healthcare, cosmetic, and other industries [1]. As sources of multiple compounds, natural products are useful against a wide range of diseases and have attracted increasing attention in recent years because of their ease of availability and fewer side effects [2].

**Figure 1:**
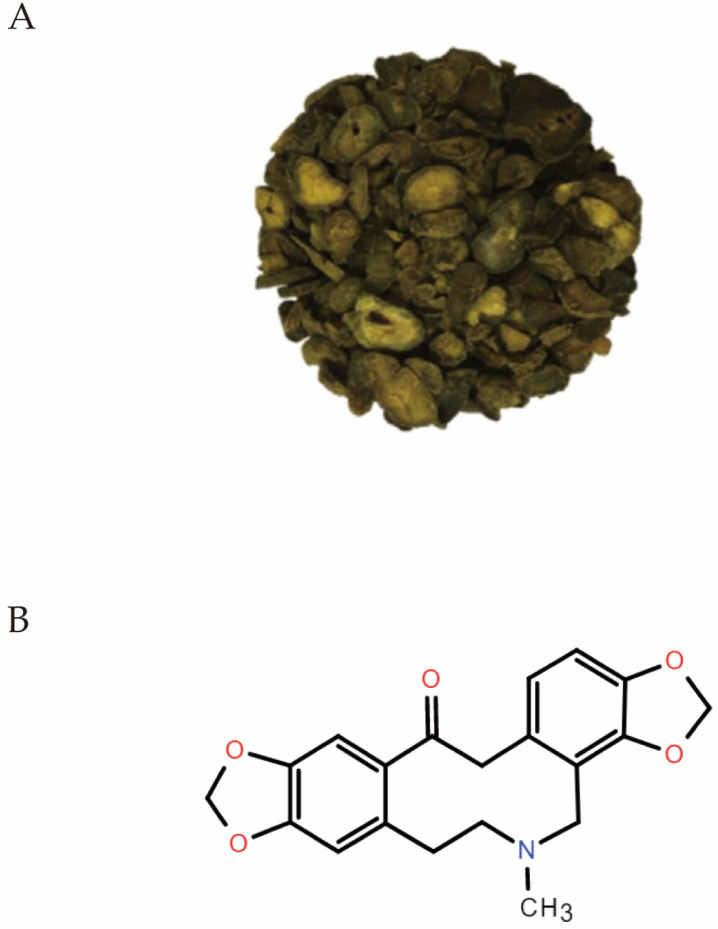
(**A**) A classic picture (reprinted from Elsevier, 131, Alam, M.B., et al., *Protopine attenuates inflammation stimulated by carrageenan and LPS* via *the MAPK/NF-kappaB pathway*, Pages 2, Copyright (2019), with permission from Elsevier) of *Corydalis yanhusuo* W.T. Wang tuber [3]. (**B**) Chemical structure of Protopine.

*Corydalis yanhusuo* W.T. Wang (Figure 1A), a valuable medicinal herb included in the Chinese Pharmacopoeia, its powder, and decoction are widely used to relieve pain and inflammation. It is still very popular today because it is beneficial for blood circulation [3]. The principal active substances of *Corydalis yanhusuo* W.T. Wang are alkaloids, which are divided into three categories: protoberberine, prototropine (protopine), and aporphine alkaloids [4].

Protopine (Figure 1B), C_20_H_19_NO_5_, is widely derived from a variety of plants, such as the Papaveraceae family (such as *Corydalis yanhusuo* W.T. Wang), Elaeocarpaceae family, and Fumariaceae family as well as Berberidaceae family and Ranunculaceae family [5]. The powdered rhizome of *Corydalis yanhusuo* was extracted with 95% alcohol five times. Then the extract was freeze dried. High performance liquid chromatography (HPLC) showed the content of protopine [6]. In addition, protopine can be synthesized based on a ring enlargement of indeno[2,1-*a*][3]benzazepines by a singlet oxygen oxygenation and conversion of an amide carbonyl group of the 10-membered keto-lactam to a methylene group and be biosynthesized from the benzyltetrahydroiso-quinoline alkaloids [7,8]. A variety of pharmacological activities of protopine have been reported [3]. However, to date, there has been no detailed review of protopine. To illustrate the identification and quantification, metabolism and pharmacokinetics, pharmacological activities, and botanical preparations for further scientific studies and clinical applications, we conducted a detailed analysis of protopine with data from 1986 to 2021.

## 2. Identification and Quantification of Protopine

With the increased research on protopine, there are more and more reports about its identification and quantification. Guo et al. developed a liquid chromatography–electrospray ionization–tandem mass spectrometry (LC-ESI-MS/MS) method, which provided a rapid, accurate and highly selective determination of protopine in rat plasma and tissues protopine [9]. Wang et al. identified protopine from Yuanhu with the help of a comprehensive 2D ATPES-decorated MCF7-CMC/capcell-C18 column/TOFMS system [10]. Other scholars reported that protopine can be identified by the LC-MS/MS with the biosynthetic pathway of isoquinoline alkaloid or the Ultrahigh-performance liquid chromatography–tandem quadrupole Exactive Orbitrap mass spectrometry (UHPLC/Q-Exactive Orbitrap MS) analysis combined with the four-step screening strategy [8,11]. In addition, protopine from the root of *Glaucium flavum* was quantified using HPLC-DAD method [12]. The concentration of protopine in methanolic extract of *Fumaria indica* was estimated by high-performance thin-layer chromatography (HPTLC) [13]. Wu et al. identified and quantified the intracellular concentration of protopine in HL-7702 cells by liquid chromatography-tandem mass spectrometry (LC-MS/MS) [14]. Protopine can also be identified and quantified by GC-MS and TLC/GC-MS methods [15,16]. Compared to HPLC-MS or HPLC-NMR, TLC/GC-MS has the advantages of short identification time, low cost of separation, and simple operation. The TLC/GC-MS is more suitable for rapid screening of a large number of small mass samples from plants containing alkaloids [16].

## 3. Metabolism and Pharmacokinetics of Protopine

**Figure 2:**
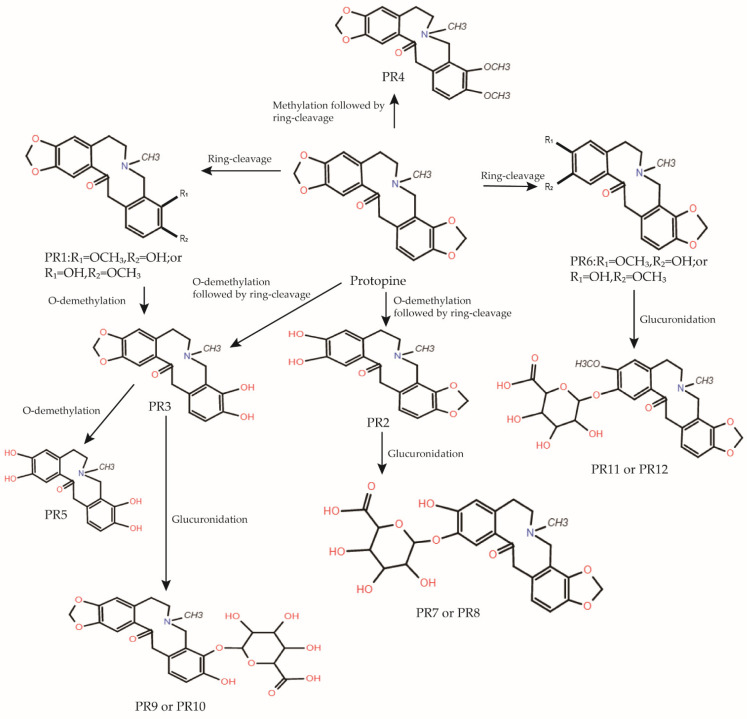
Proposed metabolic pathways of Protopine in rats in vivo [5,21].

Current pharmacokinetic studies related to protopine mainly include the metabolic studies of the *Shao-Fu-Zhu-Yu Decoction*, Yuanhu Zhitong prescription (YZP), *Chelidonium majus* L., *Rhizoma Corydalis* and *Dactylicapnos scandens*, which use protopine as a metabolite or active ingredient, and the systematic pharmacokinetic study of the monomeric protopine itself [5,9,17,18,19,20,21]. In a recent study, Huang et al. investigated the biotransformation of protopine in plasma, urine, and feces after oral administration in rats using HPLC-QqTOF-MS. The results showed gender differences in the biotransformation of protopine in urine, protopine, and its metabolites were not detected in plasma, and two metabolites of protopine, PR2 and PR6, were detected in feces. In addition, protopine is widely distributed in tissues and is present in low levels of residues. The metabolic pathways of protopine in vivo include ring cleavage, demethylation following ring cleavage, and glucuronidation. Huang et al. identified twelve metabolites of protopine in rats and proposed possible metabolic pathways for protopine in vivo shown in Figure 2 [5,21].

## 4. Pharmacological Activities of Protopine

### 4.1. Anti-Inflammatory Activities

Inflammation is an adaptive response triggered by damaging agents, such as infection and tissue damage [22]. In general, conditions that trigger inflammation can be classified as infection, tissue damage, tissue stress, and malfunction. Various conditions correspond to different physiological and pathological outcomes. When the body is infected, it reacts defensively. If the infection is not eliminated, it will lead to autoimmunity, inflammatory tissue damage, and sepsis. When tissue damage occurs, the body will repair the damaged tissue; however, if the repair is insufficient, it will lead to fibrosis, metaplasia, or tumor growth. In addition, when tissue stress and failure occur, the body creates an inflammatory response to adapt to the pressure and restore steady state conditions; otherwise, there will be changes in the steady-state set point, steady-state diseases, or the development of autoinflammatory diseases [22]. When these pathological results occur, the body must be treated with anti-inflammatory drugs to prevent further damage from inflammation. Zhang et al. explored the effects of intragastric administration of protopine on acute kidney injury (AKI) in mice using lipopolysaccharide (LPS)-induced acute kidney injury (AKI) as a model. They found that protopine significantly reduced LPS-induced levels of blood urea nitrogen (BUN), serum creatinine (Scr), inflammatory cells (neutrophils and macrophages), and inflammatory factors (IFN-γ, TNF, and IL-2, but not IL-10) by inhibiting apoptosis and the Toll-like receptor (TLR4) pathway in a dose-dependent manner (5, 15, 30 mg/kg) [23]. Alam et al. found that protopine (5, 10, 20 μM) inhibited the secretion of iNOS and COX-2 and suppressed the production of inflammatory factors (TNF-α, IL-1 β, and IL-6) through NF-κB (I κ-B) and MAPKs (including p38, ERK1/2, and JNK) pathways in an LPS-stimulated BV2 cell model. In addition, in a carrageenan (CA)-induced mouse model, oral administration of protopine (50 mg/kg) was found to inhibit the expression of iNOS and COX-2 proteins via the NF-κB pathway, thereby alleviating CA-induced paw edema in mice [3]. In LPS-induced mouse macrophage RAW264.7 cells, Bae et al. showed that protopine (20, 40 μg/mL) can reduce LPS-stimulated nitric oxide (NO), cyclooxygenase-2 (COX-2), and prostaglandin E (2) (PGE (2)) production by inhibiting NF-κB activation and phosphorylation of MAPKs [24]. Saeed et al. demonstrated that the inhibitory effect of intraperitoneal injection of protopine (50–100 mg/kg) on carrageenan-induced rat paw edema was three times higher than that of aspirin [25]. The above studies suggest that protopine can inhibit the production of inflammatory mediators by inhibiting the Toll-like receptor (TLR4), NF-κB, and MAPK pathways and apoptosis, thus achieving anti-inflammatory activity. Table 1 describes the anti-inflammatory activity of protopine mentioned above in vivo and in vivo.

### 4.2. Anti-Platelet Aggregation Activities

**Figure 3:**
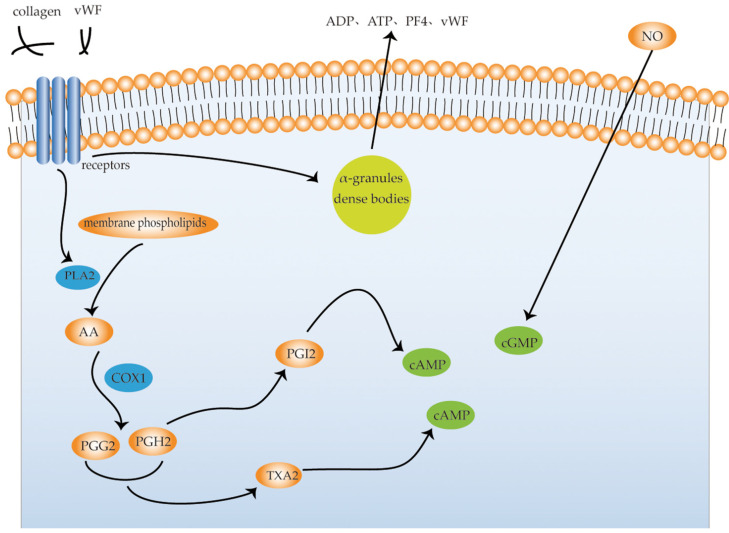
Signaling pathways of platelet activation. vWF, von Willebrand factor; PLA2, phospholipase A2; AA, arachidonic acid; PGG2, prostaglandin G2; PGH2, prostaglandin H2; PGI2, prostaglandin I2; COX1, cyclooxygenase 1; PF4, platelet factor 4.

Platelets are enucleated blood cells with a diameter of 2–4 μm generated by megakaryocytes, which maintain primary hemostasis by forming blood clots on damaged endothelial vessels [26]. Platelets in the circulating blood are generally in a “static state”. When a blood vessel is injured, platelet function is activated. The exertion of platelet function requires five basic characteristics: platelet adhesion, platelet aggregation, platelet secretion, and platelet contraction. The aggregation of platelets at the site of vascular injury is very important for thrombus formation [27]. Platelet activation is initiated by the interaction between adhesion receptors (integrin α 6 β 1, α 2 β 1, α IIb β 3, and glycoprotein (GP) Ib-IX-V complex) and their ligands, such as collagen and von Willebrand factor (vWF). When platelets are activated, Ca^2+^-dependent and protein kinase-dependent phospholipase A2 (PLA2) are subsequently activated to cleave membrane phospholipids to produce arachidonic acid (AA). AA produces prostaglandin G2 (PGG2) and prostaglandin H2 (PGH2) in the presence of cyclooxygenase 1 (COX1). The two can form TXA2 in the presence of thromboxane synthase, which reduces cAMP in platelets and strongly promotes platelet aggregation. When activated, platelets release substances stored in their dense bodies, α-granules, or lysosomes. The substances released from dense bodies are primarily ADP, ATP, 5-HT, and Ca^2+^; the substances released from α-granules, including β-thromboglobulin, platelet factor 4 (PF4), vWF, fibrinogen, coagulation factor V (FV), thrombin sensitive protein, and PDGF. ADP, 5-HT, TXA2, histamine, and thrombin, are physiological aggregates that cause platelet aggregation and play a positive role in the clotting process; conversely, prostacyclin (PGI2) and nitric oxide (NO) inhibit platelet aggregation. PGH2 produces PGI2 in the presence of prostacyclin synthase, which activates adenylate cyclase (AC), increases cAMP, and inhibits platelet aggregation, while NO activates guanylate cyclase (GC), increases cGMP, relaxes vascular smooth muscle, and inhibits platelet aggregation [28] (Figure 3). Saeed et al. investigated the effects of protopine on human platelet aggregation via cyclooxygenase. They found that protopine selectively inhibited the synthesis of thromboxane A2 (TXA2) via the COX pathway but did not affect the lipoxygenase (LOP) pathway [25]. In a rabbit platelet model, Ko et al. concluded that protopine (100 μg/mL) inhibited the formation of thromboxane and the degradation of phosphatidylinositol, thereby reducing cytoplasmic Ca^2+^ and inhibiting platelet aggregation [29]. Shen et al. found that protopine inhibited the release and influx of platelet Ca^2+^, which in turn inhibited platelet aggregation [30]. Another study found that the antiplatelet effect of protopine may be related to the increase in cyclic guanosine monophosphate (cGMP) and inhibition of the platelet active substance 5-HT release [31]. In a study on the metabolism of cAMP, it was found that protopine (0.25, 1.0 mM) inhibited platelet aggregation by increasing the activity of platelet adenylate cyclase (AC), and thus the content of cyclic adenosine monophosphate (cAMP), rather than by phosphodiesterase [32]. Shiomoto et al. found that protopine (0.25, 1.0 mM) inhibited the release of arachidonic acid (AA) and platelet-activating factors from platelets and inhibited the activity of thromboxane synthase, thereby inhibiting normal physiological activity of platelets [33]. These studies suggest that protopine inhibits platelet aggregation in different ways and holds promise for the treatment of thrombotic disorders.

### 4.3. Anti-Cancer Activity

Cancer was one of the most severe diseases in the 20th century, and its incidence is still on the rise. One in four people are at risk of developing cancer during their lifetime [34]. Cancer is characterized by six biological abilities: maintenance of proliferation signals, avoidance of growth inhibitors, resistance to cell death, immortalizing replication, induction of angiogenesis, and activation of invasion and metastasis. The study of these biological abilities will benefit the development of new approaches to human cancer treatment [35]. In recent years, an increasing number of studies have revealed the anticancer activity of natural products and their derivatives. These natural products mainly include polyphenols (such as curcumin and resveratrol), cardiotonic steroids (such as toad venom and digoxin), terpenoids (such as paclitaxel and artemisinin), and polysaccharide extracts (such as lentinan). Given the beneficial anticancer effect of natural products and their derivatives, researchers are increasingly interested in finding new potential natural products for anti-tumor therapy [36]. Protopine has also been shown to be useful for anti-cancer therapy. Table 2 describes the inhibitory effect of protopine on different tumor cells and its mechanism.

#### 4.3.1. Liver Cancer

Patients with liver cancer are often diagnosed at a late stage, resulting in poor prognosis. Over 90% of all liver cancer cases are hepatocellular carcinoma (HCC), for which chemotherapy and immunotherapy are the best treatment options. Natural compounds can provide a better prognosis for patients because of their lower systemic toxicity and fewer side effects [37]. It has been demonstrated that protopine (10, 20, 40 μM) inhibits the viability of hepatocellular carcinoma cells in a caspase-dependent manner, triggers apoptosis through endogenous pathways, and inhibits the PI3K/Akt signaling pathway by inducing intracellular ROS accumulation in vivo. Protopine (5, 10, 20 mg/kg) can inhibit tumor growth in xenografted mice without obvious toxicity in vivo [38]. By studying the effects of protopine (1, 10, 25, 50, 75 μM) on cytochrome P450 (CYPS) 1A1 and 1A2 mRNA levels, CYP1A protein and activity levels in human hepatocytes and hepatoma HepG2 cells, Jiri Vrba et al. showed that the use of products containing protopine is safe considering the possible induction of CYP1A enzymes [39]. In another study, Yu et al. found that protopine (1000 μg/mL) had antitumor activity against hepatoma HepG2 cells using an MTT anti-tumor assay [40].

#### 4.3.2. Colon Cancer

Colon cancer is a common malignancy that is often terminal, as are lung, breast, and prostate cancers [41]. Traditional cancer treatments, such as surgery, radiotherapy, and chemotherapy, have some limitations and adverse reactions; therefore, there is a constant search for new ways to treat colon cancer [42]. Recently, some scholars have found that protopine can induce apoptosis and autophagy in HCT116 colon cancer cells to exert its anti-colon cancer effect. Specifically, protopine (10, 20, 40 μM) can promote the phosphorylation of Ser residues of the tumor suppressor p53, thereby stabilizing the p53 protein and up-regulating the expression of its downstream genes p21WAF1/CIP1 and Bax, inhibiting the proliferation of colon cancer cells, activating caspase-3/7, cleaving poly ADP-ribose polymerase, and increasing the number of Annexin V-FITC-positive cells to induce apoptosis of colon cancer cells as well as promote microtubule-associated protein 1 light chain 3 (LC3) spot formation and LC3-II reversal to induce autophagy in colon cancer cells [43]. In another study, Yu et al. found that protopine (1000 μg/mL) had antitumor activity against colon adenocarcinoma SW480 cells using an MTT anti-tumor assay [40].

#### 4.3.3. Breast Cancer

According to data provided by the charity Breast Cancer Care, approximately 60,000 people are diagnosed with breast cancer each year, and the risk of developing the disease in a person’s lifetime is 1 in 8 [44]. With the continuous progress of surgical techniques, there is an increasing number of treatment options for breast cancer patients [45]. In recent years, an increasing number of natural products have been reported to have anti-breast cancer effects. Effective breast cancer treatment can provide patients with the best quality of life and minimal adverse effects [46]. Recently, Kai He et al. reported that protopine (30, 100 μM) had anti-adhesion and anti-invasive effects on breast cancer MDA-MB-231 cells and reduced the expression of epidermal growth factor receptor, intercellular adhesion molecule-1, αv-integrin, β-1-integrin, and β-5-integrin [6].

#### 4.3.4. Pancreatic Cancer

Pancreatic cancer, a fatal invasive tumor, accounts for 2% of all cancers and 5% of cancer-related deaths, occurring primarily in men and the elderly (40–85 years old). Its incidence has gradually increased over the past few years, and it is the most prevalent asymptomatic cancer [47]. With the development and advancement of surgical techniques and the medical environment, such as the introduction of laparoscopy and neoadjuvant radiotherapy and chemotherapy, the treatment of pancreatic cancer is moving forward; however, it has led to only modest improvements in treatment outcomes [48]. Recently, Garcia-Gil et al. showed that protopine/gemcitabine reduced G1, S, and G2/M phases and increased the sub-G1 phase in pancreatic cancer MIA Paca-2 and PANC-1 cells. Protopine/gemcitabine reduced the survival rate of pancreatic cancer MIA Paca-2 and PANC-1 cells and reversed the decrease in the survival rate of HDFS and tumor MCF-7 cells induced by gemcitabine. These results strongly suggest that the specific combination of (50 μM) protopine+ (50 μM) gemcitabine induces cytotoxicity in pancreatic cancer MIA Paca-2 and PANC-1 cells without significant cytotoxicity against HDFS [49].

#### 4.3.5. Prostate Cancer

Although prostate cancer has a high survival rate before metastasis, it is largely incurable even with multimodal treatment once metastasis occurs [50]. Therefore, early diagnosis and treatment are important to reduce the mortality of prostate cancer. As a novel microtubule stabilizer, protopine induces cell cycle arrest in the G2/M phase, leading to apoptosis. Phosphorylation of Thr161 and dephosphorylation of Tyr15 appear to play an important role in the activation of Cdk1. Protopine (30 μM) can induce Cdk1 phosphorylation at Thr161 and dephosphorylation at Tyr15 to activate Cdk1. The pro-apoptotic effect of Cdk1 has also been reported in many anti-tubulin drugs, including paclitaxel and vincristine. At the same time, mitochondria play a particularly important role in apoptosis. Bcl-2 family proteins are central regulators of mitochondrial-mediated cell survival and death. The phosphorylation of Bcl-2 at Ser70 may be required to enhance its anti-apoptotic function, and protopine can induce the phosphorylation of Bcl-2 Ser70 residues. Protopine (30 μM) also downregulated the expression of another member of the Bcl-2 family, Mcl-1, which plays a central role in antagonizing the apoptosis signaling pathway. These studies strongly suggest that protopine can exert its anti-prostate cancer (HRPC) cellular activity by regulating the activity of CDK1 and the apoptotic pathway of the Bcl-2 protein family [51].

#### 4.3.6. Lung Cancer

Lung cancers are classified as small cell carcinoma and non-small cell carcinoma (such as adenocarcinoma, squamous cell carcinoma, and large cell carcinoma), and these classifications are used to determine treatment and prognosis [52,53]. Despite significant advances in the diagnosis and treatment of lung cancer over the past 25 years, the prognosis remains poor for all but the most localized lung cancers [54]. In an anti-tumor assay using MTT, Yu et al. found that protopine (100 μg/mL) had anti-tumor activity against lung cancer A549 cells [40].

### 4.4. Analgesic Activity

Until the 1960s, pain was considered an inevitable sensory response to tissue damage [55]. Pain is major medical, social, and economic problem [56]. With the rapid advancement of medical science and technology, an increasing number of natural plant extracts have been used for pain relief owing to their efficiency and safety [57]. *Corydalis yanhusuo* is the most effective painkiller, known as the “morphine” of traditional Chinese medicine, and its component protopine has recently been found to have analgesic effects. Xu et al. found that intracerebroventricular injections of protopine (20–200 μg/mouse) in mice had a significant analgesic effect, which was completely blocked by naloxone, an opioid receptor blocker. CaCl_2_ or methotrexate completely blocked the analgesic effect of protopine, while nifedipine, a Ca^2+^ channel blocker, could enhance the analgesic effect. Similarly, reserpine and the α-adrenoceptor blocker phentolamine diminished the analgesic effect of protopine, whereas the β-adrenoceptor blocker propranolol did not affect its analgesic effect. The above experiments suggest that protopine exerts its analgesic activity mainly through the activation of opioid receptors, inhibition of calcium ions, and activation of alpha receptors [58]. As common analgesic targets, voltage-gated sodium channels (VGSCs) play an important role in the generation and conduction of action potential. Different subtypes of VGSCs are associated with different pathological processes, and Nav1.7 is closely associated with pain triggering. NaV1.5 is widely distributed in the heart and is regarded as an important target for screening cardiac risk. Xu et al. investigated the effects of protopine (10 μM) on Nav1.7 and NaV1.5 in vivo using a patch-clamp recording technique. The analgesic activity of protopine (10, 20, 40 mg/kg) in vivo was also verified using a formalin-induced pain model. The results showed that protopine significantly inhibited Nav1.7 and increased the level of CK-MB, a highly specific and sensitive marker of cardiomyocyte death, indicating that the analgesic activity of protopine depends mainly on the regulation of Nav1.7, but at the same time, it poses a significant risk of cardiac damage via Nav1.5 [57].

### 4.5. Vasodilatory Activity

The vasodilating effects of protopine have been reported in the last century. Ko et al. studied the vasodilatory effects of protopine using isolated rat thoracic aorta as a model. Protopine inhibited norepinephrine (NE, 3 µM)-induced tense constriction of rat thoracic aorta and high K^+^ (60 mM)-induced calcium-dependent constriction of rat aorta. The mechanism revealed that protopine (25, 50 μg/mL) inhibited NE-and K^+^-induced Ca^2+^ influx but had no effect on cAMP and cGMP levels. It has been suggested that protopine plays a role in vasodilation through the inhibition of Ca^2+^ influx [59]. However, Huang et al. found that protopine had a vasodilation effect on rabbit thoracic aorta, mesenteric artery, and portal vein by inhibiting intracellular calcium release but not calcium influx [60]. Another study found that in the presence of noradrenaline (NA), protopine (100 μM) promoted the transfer of PKC from the cytoplasm to the cell membrane and induced vasodilation by reducing Ca^2+^ and increasing cAMP and cGMP [61].

### 4.6. Anticholinesterase Activity

Cholinesterase (ChE) is divided into acetylcholinesterase (AChE, also known as true cholinesterase) and butyrylcholinesterase (BChE, also known as pseudocholinesterase) [62]. Alzheimer’s disease (AD) can be divided into primary dementia, vascular dementia, and a combination of the two. The former is a highly age-related degenerative disease of the central nervous system with progressive cognitive impairment and memory deficits. With the extension of human life expectancy and the increasingly prominent problem of social aging, the number of AD patients will continue to increase. Acetylcholine deficiency is closely associated with AD Various cholinesterase inhibitors have been developed to treat AD, including natural inhibitors and synthetic analogs [63]. Kim et al. found that protopine inhibited AChE activity in a dose-dependent manner [64]. Another study concluded that protopine inhibited AChE activity in a dose-dependent manner (50, 100 μM). This study also found that the anti-acetylcholinesterase activity of protopine was specific, reversible, and competitive. Moreover, intraperitoneal injection of protopine (0.1, 1 mg/kg) significantly reduced the scopolamine-induced memory impairment in mice. This supports the theoretical feasibility of protopine for the treatment of AD [65].

### 4.7. Anti-Addictive Activity

Opioids are one of the most clinically effective painkillers; however, the abuse of opioids is on the rise worldwide. The primary reason for limiting their long-term use is due to the potential for addiction [66]. As early as the end of the last century, protopine (5, 10, 50 μM) was shown to reduce morphine withdrawal in a concentration-dependent manner through the anticholinergic pathway. This was also the first demonstration of the anti-opioid addictive properties of protopine [67]. In addition, experimental studies by Capasso et al. have also shown that protopine significantly reduces morphine withdrawal in a concentration-dependent manner [68]. These studies suggest that protopine may be a potential anti-addictive drug.

### 4.8. Anticonvulsant Activity

Epilepsy is a brain disorder characterized by a persistent tendency to generate epileptic seizures [69]. It is one of the most common chronic neurological diseases worldwide. Many drugs used to treat epilepsy have adverse side effects and drug interactions. As a result, there has been a search for alternative anticonvulsant drugs [70]. In a pentylenetetrazol-induced seizure mouse model, with sodium valproate as the reference, protopine showed some anticonvulsant activity, significantly prolonged the latency of seizures, shortened the duration of convulsions, and significantly reduced the severity of convulsions. In addition, the anticonvulsant effect of protopine (0.005, 0.05 mg/kg) was dose-dependent [71]. Kardos et al. found that protopine (0.1, 1, 10 μM) enhanced the binding of 3H-gamma-aminobutyric acid (3H-GABA) to rat brain synaptic membrane receptors, increased GABA-mediated Cl^−^ influx, led to membrane hyperpolarization, decreased membrane excitability, and exerted its sedative and anticonvulsant effects [72].

### 4.9. Antipathogen Activity

#### 4.9.1. Helicobacter Pylori

*Helicobacter pylori* is the most common pathogen in the world’s population. It is the primary risk factor for gastric ulcers, duodenal ulcers, and gastric cancer [73]. There is growing evidence that natural plant extracts are the preferred choice for the development of treatments and prevention of *Helicobacter pylori* infection. Mahady et al. found that protopine (MIC_50_ = 100 μg/mL, MIC_90_ > 100 μg/mL) inhibits the growth of *Helicobacter pylori* in vivo [74]. Table 3 summarizes the action mechanism of Protopine against different pathogens.

#### 4.9.2. Parasite

##### Leishmania

Leishmaniasis is a zoonotic disease caused by *Leishmania protozoa* and is transmitted between arthropods and mammals. Leishmania parasites in visceral macrophages can cause visceral leishmaniasis (VL). Visceral leishmaniasis, one of the deadliest parasitic diseases in the world, kills more than 50,000 people each year. In 2015, the World Health Organization (WHO) classified VL as a neglected tropical disease (NTD), prompting intensive research [75]. Protopine blocks the function of UDP- galactosidase (UGM), which is the key enzyme for cell wall synthesis in *Leishmania protozoa*, and therefore plays a role in anti-leishmaniasis and could be used in drug design [76].

##### Strongyloides

Strongyloidiasis is a parasitic disease with a worldwide distribution, and an estimated 614 million people are infected. Strongyloidiasis is usually asymptomatic or has only mild skin, respiratory, or gastrointestinal symptoms. Therefore, it is easy to cause neglect, leading to exacerbation of the infection and even death in patients with poor immune status [77,78]. One study using infective third-stage larvae of *S. ratti* and *S. venezuelensis* as a model for pathogenic nematodes showed that protopine had strong nematocidal activity with low cytotoxicity [79].

#### 4.9.3. SARS-CoV-2

The novel coronavirus *SARS-CoV-2*, named COVID-19 by the World Health Organization (WHO) on 11 February 2020, is a highly transmissible and pathogenic coronavirus [80,81]. Coronavirus disease (COVID-19) is spreading worldwide. Many potential drugs against COVID-19 have been identified, including small molecular drugs, interferon therapy, vaccines, oligonucleotides, peptides, and monoclonal antibodies [82]. RNA-dependent RNA polymerase is essential for the survival of RNA viruses. RNA is synthesized by polymerase according to the mechanism of primer independence and primer dependence in an RNA virus. An increasing number of antiviral drugs are being manufactured to treat diseases caused by ribonucleic acid viruses based on RNA-dependent RNA polymerase. Pandeya et al. used molecular docking analysis to show that protopine may be a potential RNA-dependent RNA polymerase inhibitor of *SARS-CoV-2* [83]. However, this is only a theoretical computational study, and further experiments in vivo and in vivo are needed to prove the pharmacological activity of protopine against *SARS-CoV-2*.

### 4.10. Antioxidation Activity

Oxidative stress occurs when the production of reactive oxygen species/nitrogen species exceeds consumption [84]. Oxidative stress is the negative effect of free radicals within the body and is considered an important contributor to aging and disease. Oxidative stress is closely associated with age and plays an important role in the occurrence and development of age-related chronic diseases such as diabetes and cardiovascular, renal, lung, and skeletal muscle diseases [85]. Some scholars developed a focal cerebral ischemia model of middle cerebral artery occlusion in rats by thread occlusion, and these rats were randomly divided into two groups. Intraperitoneal injection of protopine pretreatment reversed serum superoxide dismutase activity in a dose-dependent manner (1.96, 3.92 mg/kg) in rats with cerebral ischemia, reduced the infarct rate and serum lactate dehydrogenase activity induced by middle cerebral artery occlusion, and improved neurological deficit scores and brain histological changes in rats [86]. In addition, the antioxidant effects of protopine were observed in H_2_O_2_-induced injury in PC12 cells. Protopine (5, 50 μM) enhanced the activities of superoxide dismutase, glutathione peroxidase, and catalase and inhibited the increase in intracellular Ca^2+^, the expression of caspase-3, and apoptosis induced by H_2_O_2_ [87].

### 4.11. Hepatoprotective Activity

In recent decades, drug-induced liver injury has become the leading cause of acute liver disease, which poses a great challenge to clinicians and researchers and has attracted great attention [88]. However, the patient’s condition necessitates the use of hepatotoxic drugs. Some studies have shown that protopine has hepatoprotective effects. Both paracetamol and carbon tetrachloride have obvious hepatotoxicity, resulting in significantly elevated serum AST and ALT levels in rats. Protopine (11 mg/kg) can protect the liver from hepatotoxicity induced by paracetamol and carbon tetrachloride by inhibiting microsomal drug-metabolizing enzymes (MDME) [89].

### 4.12. Neuroprotective Activities

Eighty percent of stroke patients are caused by cerebral ischemia and the other twenty percent are caused by cerebral hemorrhage [90]. Calcium overload, apoptosis, inflammation, and glutamate excitotoxicity are involved in the pathophysiological process after stroke. Using a model of focal cerebral ischemia, Xiao et al. found that intraperitoneal injection of protopine (1.96, 3.92 mg/kg) pretreatment decreased serum lactate dehydrogenase activity, total calcium levels, and the number of TUNEL-positive cells in the penumbra in a dose-dependent manner, increased serum superoxide dismutase activity, and improved neurological deficit scores and brain histological changes. However, since calcium antagonism can also cause anti-apoptotic and antioxidant effects, the exact neuroprotective mechanism of protopine needs to be further investigated [86].

### 4.13. Cytotoxic and Anti-Proliferative Activities of Protopine

The cytotoxicity of protopine has been shown in a wide variety of tumor cell lines. Protopine exhibited significant growth inhibitory activity in vivo against HL-60, A-549, and MCF-7 cancer cell lines with IC50 values of 6.68, 20.47, and 22.59 μM, respectively [91]. In addition, an MTT assay also confirmed the cytotoxic effect of protopine on HepG2, SW480 and A549 tumor cells [40], as well as MIA PaCa-2, PANC-1, U343, U87, and MCF-7 tumor cells [49]. Protopine (20–40 μM) showed moderate cytotoxicity against human breast cancer cells MDA-MB-231, while it had no effect on normal human cell (HUVEC) viability; however, high concentrations of protopine (>40 μM) may promote breast cancer cell viability by similar anti-apoptotic and/or antioxidant mechanisms [12].

The anti-mitotic effect of protopine (30 μM) has also been reported. It may exert an anti-proliferative effect by inducing microtubule protein polymerization, blocking mitosis, and ultimately leading to apoptosis in human hormone-independent prostate cancer (HRPC) cells [51]. In another study, it was found that protopine may exert an antimitotic effect by altering microtubule dynamics without affecting microtubule protein polymerization [92].

## 5. Botanical Preparations of Protopine

Protopine is believed to be an active constituent of many botanical preparations, such as *Chelidonium majus* L., *Fumaria* L., *Sanguinaria canadensis*, *Corydalis Calliantha* Long, *Dactylicapnos scandens*, *Corydalis yanhusuo* W.T. Wang, *Hypecoum erectum* L., *Macleaya cordata*, and *Nandina domestica* [3,9,24,93,94,95,96,97,98]. Table 4 illustrates the application in diseases of these botanical preparations containing protopine.

## 6. Conclusions

Protopine is an alkaloid found in *Corydalis yanhusuo* and is gaining attention as research on natural products has intensified. There are also many botanical preparations containing protopine used in veterinary and human medicine in (Table 4). It has a wide range of sources, such as plants, organic synthesis, and biosynthesis. A large number of methods have proved suitable for the rapid identification and quantification of protopine, which has twelve metabolites in vivo and its possible metabolic pathways have been proposed (Figure 2). Several of its potential pharmacological activities have been reported. Protopine exerts an anti-inflammatory activity by inhibiting the Toll-like receptor (TLR4), NF-κB, and MAPK pathways, as well as apoptosis. Protopine can increase the levels of cAMP and cGMP, inhibit the release of AA and platelet-activating factors, and the activity of thromboxane synthase, thus inhibiting platelet aggregation and playing an anticoagulant effect. Protopine also showed anticancer effects on tumor cells of liver cancer, colon cancer, breast cancer, pancreatic cancer, prostate cancer, and lung cancer. Protopine can also induce analgesia by activating opioid receptors, α-receptors, and inhibiting calcium ions, the voltage-gated sodium channel subtype Nav1.7. In addition, the vasodilation effect of protopine has been reported, but its specific mechanism remains controversial. This alkaloid also antagonizes the activity of acetylcholinesterase in a specific, reversible, and competitive manner, which is expected to play a therapeutic role in Alzheimer’s disease. Furthermore, protopine significantly reduced morphine withdrawal in a concentration-dependent manner, displaying its potential as an anti-addiction drug. Protopine also exerts sedative and anticonvulsant effects by enhancing the binding of 3H-gamma-aminobutyric acid (3H-GABA) to rat brain synaptic membrane receptors. In addition, it can also inhibit or eliminate pathogens such as Helicobacter pylori, Leishmania, Strongyloides, and SARS-CoV-2. In addition, protopine exhibited hepatoprotective activity. Protopine improved neurological deficit scores and brain histological changes in a dose-dependent manner, but its specific mechanism has not been fully elucidated. Finally, it exhibits toxic activity on a variety of tumor cells and also has anti-mitotic effects, but its mechanism is still controversial, and further research is needed. In summary, protopine is available from multiple sources, can be rapidly identified, and has a wide range of pharmacological activities, showing the feasibility of potential drug therapy for a variety of diseases; however, its pharmacokinetics in vivo is poorly studied, and some mechanisms of pharmacological activity remain controversial and unclear, and further experiments and more comprehensive and reliable data in vivo and in vivo are needed to elucidate, which will be the focus of future researches of protopine. Therefore, our study may give some foundation for further research on protopine and even its clinical application.

## Figures and Tables

**Table 1:** Anti-inflammatory activities of protopine.

In Vivo/In Vivo	Signaling Pathway	Effects	References
LPS-induced AKI mouse model	apoptosis and TLR4	reduces levels of BUN, Scr, inflammatory cells, and inflammatory factors (IFN-γ, TNF, and IL-2)	[7]
PS-stimulated BV2 cell model	NF-κB andMAPK	inhibits the production of iNOS, COX-2, and inflammatory factors (TNF-α, IL-1 β, and IL-6)	[3]
CA-induced mouse model	NF-κB	inhibits the expression of iNOS, COX-2, and alleviated paw edema	[3]
LPS-stimulated RAW264.7 cell model	NF-κB andMAPK	reduces the production of NO, COX-2 and PGE2	[8]
CA-induced rat model		inhibits paw edema	[3]

**Table 2:** Anti-cancer activities of protopine.

Cancer Type	Cell Line	Mechanism	References
Liver Cancer	HepG2		[23]
	HepG2 and Huh-7	es regulates ROS/PI3K/Akt signaling pathway and the intrinsic pathway	[21]
Colon Cancer	HCT116	induces apoptosis and autophagy by stabilizing p53	[26]
	SW480		[23]
Breast Cancer	MDA-MB-231	anti-adhesion and anti-invasive	[30]
Pancreatic Cancer	MIA Paca-2 and PANC-1		[33]
Prostate Cancer	HRPC	regulates Cdk1 activity and Bcl-2 family of proteins.	[35]
Lung Cancer	A549_		[23]

**Table 3:** Antipathogen activities of protopine.

	Pathogen	Mechanism	References
Bacteria	*Helicobacter pylori*		[58]
Parasite	*Leishmania*	inhibits the function of UDP- galactosidase (UGM)	[60]
	*Strongyloides*		[63]
Virus	*SARS-CoV-2*	inhibits the activity of RNA-dependent RNA polymerase	[67]

**Table 4:** Application in diseases of the botanical preparations containing protopine.

	Application in Diseases	References
*Chelidonium majus* L.	Liver Dysfunction and Skin Diseases	[93]
*Fumaria* L.	Hypertension, Rash and Arthritis	[94]
*Sanguinaria canadensis*	Asthma, Tuberculosis and Dysentery	[95]
*Corydalis Calliantha* Long	Malaria	[96]
*Dactylicapnos scandens*	Hypertension, Inflammation, and Pain	[9]
*Corydalis yanhusuo* W.T. Wang	Backache, Arthralgia and Trauma	[3]
*Hypecoum erectum* L.	Inflammation	[24]
*Macleaya cordata*	Inflammation	[97]
*Nandina domestica*	Chronic Bronchitis	[98]

## Data Availability

Not applicable.
